# Patterns of multimorbidity in older adults with multiple myeloma: An analysis of SEER-Medicare

**DOI:** 10.1371/journal.pone.0330331

**Published:** 2025-08-20

**Authors:** Atinuke G. Oyinbo, Mayra Tisminetzky, Jonggyu Baek, Maira A. Castaneda-Avila, Clark DuMontier, Muthalagu Ramanathan, Kate L. Lapane, Mara M. Epstein

**Affiliations:** 1 Division of Epidemiology, Department of Population and Quantitative Health Sciences, University of Massachusetts Chan Medical School, Worcester, Massachusetts, United States of America; 2 Division of Health Systems Science, Department of Medicine, University of Massachusetts Chan Medical School, Worcester, Massachusetts, United States of America; 3 Division of Geriatric Medicine, Department of Medicine, University of Massachusetts Chan Medical School, Worcester, Massachusetts, United States of America; 4 Division of Biostatistics and Health Services Research, Department of Population and Quantitative Health Sciences, University of Massachusetts Chan Medical School, Worcester, Massachusetts, United States of America; 5 VA Boston Healthcare System and Brigham and Women’s Hospital, Boston, Massachusetts, United States of America; 6 Division of Hematology-Oncology, Department of Medicine, UMass Memorial Medical Center, University of Massachusetts Chan Medical School, Worcester, Massachusetts, United States of America; The University of the West Indies, JAMAICA

## Abstract

**Objective:**

Multimorbidity influences the management of symptoms, treatment, and health outcomes for older adults with multiple myeloma. Our study characterized patient profiles defined by discrete chronic condition patterns and examined the distribution of their sociodemographic factors in older adults at time of diagnosis with multiple myeloma.

**Methods:**

Using the Surveillance, Epidemiology and End Results (SEER) Program database linked with Medicare insurance claims (2007–2017), we examined 11,926 individuals diagnosed with multiple myeloma at age ≥ 65 years. Hierarchical cluster analysis was conducted to identify prevalent patterns of multimorbidity from thirty-three chronic conditions, and sociodemographic factors associated with the identified patterns were examined.

**Results:**

The median age of the cohort was 77 years and 48% were women. Three patient groups with distinct patterns of multimorbidity were identified: minimal multimorbidity featuring minor burden of comorbid conditions (54.3%), psychiatric and musculoskeletal multimorbidity (21.5%), and a high multimorbidity group featuring cardiometabolic and multiple system disorders (20.7%). The remaining 3.5% did not have multimorbidity. Patients in the cardiometabolic and multisystem multimorbidity pattern were the oldest, more likely to reside in geographic areas with high poverty levels and comprised more men and Hispanic individuals than the other two patterns. The psychiatric and musculoskeletal multimorbidity pattern comprised the largest proportion of women and lowest proportion of non-Hispanic Black individuals. Those with relatively minimal multimorbidity burden were the most likely to be married.

**Conclusions:**

Identifying patterns of multimorbidity in older adults newly diagnosed with multiple myeloma provides insight into the burden and clustering tendency of chronic conditions within similar patient groups. Our findings can inform tailored disease management and treatment programs that take these unique multimorbidity profiles into account.

## Introduction

Multiple myeloma accounts for 19% of new hematologic cancer cases in the United States [1]. Newly affecting over 35,780 individuals in 2024 [1], multiple myeloma is diagnosed via biomarker parameters (SLiM criteria) [2], and features of end-organ damage including hypercalcemia, renal insufficiency, anemia, and lytic bone lesions (CRAB criteria) [3]. While the incidence of multiple myeloma has risen over the past few decades, the 5-year relative survival rates have also steadily increased due to the availability of improved and novel treatment methods [4]. The majority of patients diagnosed with this condition are older adults, with a median age at diagnosis of 69 years, and two-thirds of all diagnoses occurring in adults aged ≥65 years [5]. Multimorbidity (defined as two or more coexisting chronic conditions) [6] is commonly experienced by adults aged ≥65 years [7]. Forty to sixty percent of people with multiple myeloma are affected by other chronic health conditions [8–10]. In individuals with multiple myeloma, multimorbidity is a significant predictor of poor survival [9,10] and is linked to functional status decline, poor quality of life, and treatment toxicities [11–14]. Understanding the underlying patterns of multimorbidity in this population is key to understanding how unique patterns of chronic conditions affect the prognosis and treatment trajectory of patients with multiple myeloma.

Multimorbidity is often measured using counts of chronic conditions or with indices specific to the diagnostic criteria of multiple myeloma [8,15–17]. Count-based methods may not account for the complex manifestation of multiple chronic conditions [18,19] and mainstream comorbidity indices may be limited in their ability to granularly examine multimorbidity in older adults [20], as they include a limited number of chronic conditions and may be designed to predict specific outcomes. The need for multimorbidity assessments that go beyond the prognostic significance of myeloma-related chronic diseases is crucial, given that the prevalence of multimorbidity increases with age and older adults with multiple myeloma are likely living with comorbid diagnoses beyond those directly related to the cancer. The presence of specific comorbid conditions, in addition to the total burden of multimorbidity, may further complicate and adversely impact cancer treatment selection and prognosis.

To date, one study has examined multimorbidity in older US veterans with multiple myeloma using latent class analysis [21], although this research has not been replicated in more broadly generalizable populations. Another study investigated multimorbidity patterns in older adults with monoclonal gammopathy of undetermined significance (MGUS), which is a precursor to multiple myeloma [22]. There is minimal evidence to suggest which subgroups are more likely to experience certain chronic condition groupings within the general US population of patients with multiple myeloma. The aim of this study was to identify patterns of multimorbidity prevalent upon multiple myeloma diagnosis in older adults using hierarchical cluster analysis. This study also examines the association between patient characteristics and identified multimorbidity patterns.

## Materials and methods

### Data source

The Surveillance, Epidemiology, and End Results Program data linked with Medicare claims (SEER-Medicare) consists of population-based data from cancer registries covering 48% of the US population, linked to patient-level Medicare enrollment and claims information for Medicare fee-for-service beneficiaries [23,24]. Medicare is the federal health insurance program for individuals aged 65 or older in the US. All eligible older adults are automatically enrolled into the program, which provides access to inpatient services, outpatient care, and drug prescriptions. These healthcare encounters are documented as medical claims for reimbursement to providers [25]. Over 95% of individuals aged ≥65 years who are included in SEER have been linked to Medicare enrollment data [26]. This database includes healthcare services, demographics, cancer-specific information, and chronic conditions for Medicare-insured older adults diagnosed with cancer. The University of Massachusetts Chan Medical School Institutional Review Board (IRB) approved this study (IRB number H00022359). The SEER-Medicare data are deidentified, and were first accessed 8th February 2021.

### Study population

The inclusion criteria comprised individuals who were: (1) newly diagnosed between 2007 and 2017 with multiple myeloma determined via histology code, (2) aged ≥65 years at time of diagnosis, (3) and continuously enrolled in Medicare fee-for-service for 12 months before diagnosis. Participants were excluded if they had prior cancer diagnoses, or if their multiple myeloma diagnosis was ascertained from autopsy or death certificate. The enrollment criteria were applied to ensure enough time prior to the cancer diagnosis to determine baseline chronic conditions.

### Study variables

#### Co-morbid chronic conditions.

The presence of chronic conditions at time of multiple myeloma diagnosis was determined using non-cancer chronic condition flags defined using validated algorithms in the Centers for Medicare & Medicaid Services (CMS) Chronic Conditions Data Warehouse (CCW). The operational definitions of non-cancer chronic conditions were specifically designed to identify these conditions using Medicare claims data [27]. The list of conditions used in this study included anemia, chronic kidney disease, liver disease, cardiovascular (acute myocardial infarction, atrial fibrillation, congestive heart failure, hypertension, ischemic heart disease, peripheral vascular disease, and stroke), metabolic (acquired hypothyroidism, diabetes, hyperlipidemia, and obesity), neurological and psychiatric (Alzheimer’s disease and related disorders or senile dementia, anxiety, depressive disorders, epilepsy, and schizophrenia and other psychotic disorders), respiratory (asthma and chronic obstructive pulmonary disease), musculoskeletal and pain-related (fibromyalgia, hip/pelvic fracture, mobility impairment, osteoporosis, rheumatoid arthritis/osteoarthritis, and spinal injury), sensory (glaucoma, cataract, and deafness or hearing impairment), substance use (drug use and tobacco use disorders), and skin conditions (pressure ulcers). The CCW algorithms search for condition-specific claims criteria within a 1, 2 or 3-year look back time period before a given index year and flags that condition as being present during that index year [27]. We searched for chronic condition flags in the year before and the year of the diagnosis of multiple myeloma to satisfy the criterion of an individual having that chronic condition at diagnostic baseline. Patients who did not have sufficient Medicare coverage during the lookback period were excluded, as gaps in coverage do not allow for accurate capture of pre-existing chronic conditions [28]. Patients were considered to have multimorbidity at the time of diagnosis with multiple myeloma if two or more chronic conditions were flagged according to the CCW algorithms.

#### Demographic information.

From the SEER-Medicare database, we identified information on patients’ sex, age at multiple myeloma diagnosis, race and ethnicity, marital status at diagnosis, area of residence and percent of residents living below the federal poverty line in patients’ residential area based on census tract. All of these variables were extracted from cancer registry data and were selected for their established associations with various patterns of multimorbidity in older adult populations [29].

### Statistical analysis

We used an agglomerative hierarchical cluster analysis (HCA) to identify distinct groups of patients based on similar sets of comorbid chronic health conditions. This method is appropriate for when the cutoff point for the number of clusters is determined subjectively [30]. Conditions with a prevalence ≥2% in the cohort were applied to the HCA [31], and 33 CCW conditions met this threshold. The HCA assigns individuals to clusters based on the similarity of their profiles of chronic conditions, via a clustering algorithm. The similarity between chronic conditions was measured using the Jaccard coefficient, which is an appropriate metric for clinical conditions, as it focuses only on conditions that two individuals have in common while disregarding conditions that are present in neither person [32]. The data were randomly split into two datasets with the same sample size (training and validation), and we ran the Ward clustering algorithm [33] on both separately using the ‘*hclust*’ package in R (version 4.4.0). The optimal number of multimorbidity clusters was determined by both qualitatively consulting clinicians and quantitatively examining an exhaustive list of statistical indices that deduce the best clustering solution using the ‘*NbClust*’ package from R [34]. Results from the HCA were output into a dendrogram from which the clusters were then extracted. As cluster membership was similar between the training and validation datasets, we used the Ward method for the entire study sample to improve the efficiency of the estimates.

To describe and define the conditions most characteristic of each given pattern of multimorbidity, we considered conditions with the highest observed/expected [O/E] ratios (disease prevalence in the cluster divided by disease prevalence in the entire cohort) and exclusivity (number of patients with the condition in the cluster divided by total number of patients with the condition). The O/E ratio helps highlight conditions that are overrepresented within a cluster, denoting a higher prevalence than what would be expected by chance in the study population. Exclusivity underlines which conditions uniquely characterize any given cluster compared to others. A chronic condition was deemed relevant to a given cluster based on an O/E ratio >1 and exclusivity of >25% [35–37]. Confidence intervals were derived for the O/E ratios [38]. We described the prevalence of the defining chronic conditions within each cluster and described the multimorbidity patterns by cohort characteristics. Associations between each multimorbidity pattern and demographic factors were examined using chi-square tests and the Brown-Mood median test. A sensitivity analysis was conducted by comparing results from using the Ward algorithm to that of the flexible beta algorithm. SAS version 9.4 (SAS Institute Inc., Cary, NC, USA) was used jointly with R version 4.4.0 to complete all analyses.

## Results

### Study population

**Fig 1:**
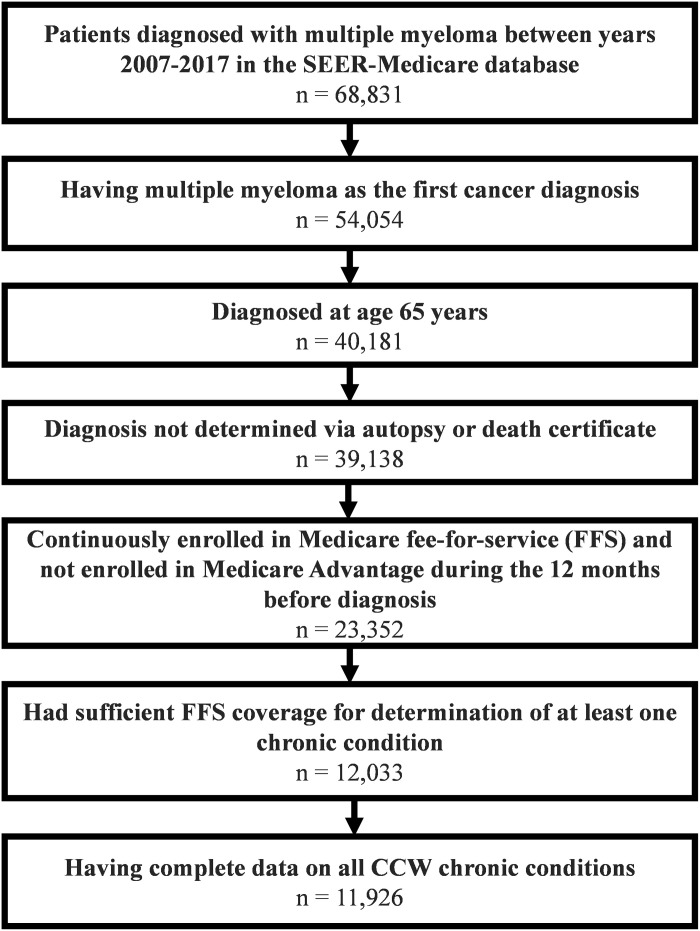
Study sample selection flowchart.

There were 11,926 eligible Medicare beneficiaries in the study cohort (Fig 1). The median age at diagnosis for patients was 77 years (Interquartile range [IQR]: 72–82 years) and 48% were women. The cohort consisted of 13.7% non-Hispanic Black individuals, as well as 6.2% Hispanic, 76.1% Non-Hispanic White, and 3.9% Non-Hispanic Asian or Pacific Islander and American Indian/Alaska Native individuals (Table 1). Nearly all (96.5%) had multimorbidity (Table 2).

**Table 1:** Distribution of sociodemographic characteristics according to multimorbidity patterns identified among 11,926 older adults diagnosed with multiple myeloma in the SEER-Medicare database, 2007–2017.

Patient characteristics (column %)	Total population (n = 11,926)	≤ One chronic condition (n = 415)	Relatively minimal (n = 6,481)	Psychiatric and musculoskeletal (n = 2,563)	Cardiovascular and multisystem (n = 2,467)	*P*
**Median age at diagnosis (IQR)**	77 (72–82)	74 (70–79)	76 (71–82)	76 (71–82)	79 (74–84)	<.0001
**Women**	48.2	28.7	45.1	65.4	41.9	<.0001
**Race/ethnicity** ^**†**^						<.0001
Hispanic	6.2	5.9	6.4	5.2	6.9	
Non-Hispanic White	76.1	77.0	73.9	83.6	74.1	
Non-Hispanic Black	13.7	14.4	15.4	8.1	15.1	
Non-Hispanic API, AI/AN	3.9	2.7	4.3	3.1	3.9	
**Marital status at diagnosis**						<.0001
Single (never married)	5.8	6.3	5.6	6.1	5.8	
Married/Partnered	38.1	40.7	40.2	35.9	34.6	
Separated/Divorced	5.4	7.7	5.3	5.3	5.5	
Widowed	17.6	10.1	16.0	20.4	20.1	
Unknown marital status	33.1	35.2	32.9	32.2	34.0	
**Census tract location** ^**†**^						0.02
Metropolitan	86.1	83.7	86.7	86.3	85.3	
Urban/Rural	13.9	16.1	14.0	15.0	12.2	
**Census tract poverty level** ^**††**^						0.01
0%–< 5% poverty	23.3	23.4	22.9	24.0	23.6	
5%–< 10% poverty	25.5	26.3	26.0	25.6	24.2	
10%–< 20% poverty	25.9	25.3	26.1	25.8	25.5	
20%–100% poverty	18.6	17.3	18.5	16.7	21.0	
Unknown poverty level	6.7	7.7	6.4	7.9	5.7	

**Notes.** IQR: Interquartile Range, API, AI/AN: Asian or Pacific Islander, American Indian/Alaska Native.^**†**^ Missing data for race/ethnicity = 78, and missing data for location = 5. ^**††**^

**Table 2:** Prevalence of thirty-three chronic conditions in older adults with multiple myeloma at diagnosis, as defined by the chronic conditions data warehouse.

Condition group	%
**Cardiovascular conditions**	
Acute myocardial infarction	2.7
Atrial fibrillation	17.7
Congestive heart failure	36.6
Hypertension	81.1
Ischemic heart disease	48.2
Peripheral vascular disease	18.3
Stroke	7.4
**Metabolic conditions**	
Acquired hypothyroidism	19.3
Diabetes	35.2
Hyperlipidemia	57.9
Obesity	10.4
**Neurological and psychiatric conditions**	
Alzheimer’s disease and related dementias	15.5
Anxiety	13.2
Depressive disorder	21.3
Epilepsy	2.1
Schizophrenia and other psychotic disorders	3.8
**Respiratory conditions**	
Asthma	7.4
Chronic obstructive pulmonary disease	19.2
**Musculoskeletal and pain-related conditions**	
Fibromyalgia	21.7
Hip/Pelvic fracture	3.8
Mobility impairments	4.6
Osteoporosis	17.4
Rheumatoid arthritis/osteoarthritis	47.9
Spinal injury	2.3
**Hematologic, kidney, and liver conditions**	
Anemia	84.7
Chronic kidney disease	54.1
Liver disease	7.2
**Sensory conditions**	
Cataract	21.1
Glaucoma	12.1
Deafness	6.9
**Substance use conditions**	
Drug use disorders	2.3
Tobacco use disorders	6.2
**Skin conditions**	
Pressure ulcers	9.9

**Notes.** Individuals can appear more than once as the conditions are not mutually exclusive. There were 3.5% of patients who did not have a chronic health condition.

### Multimorbidity patterns

**Fig 2:**
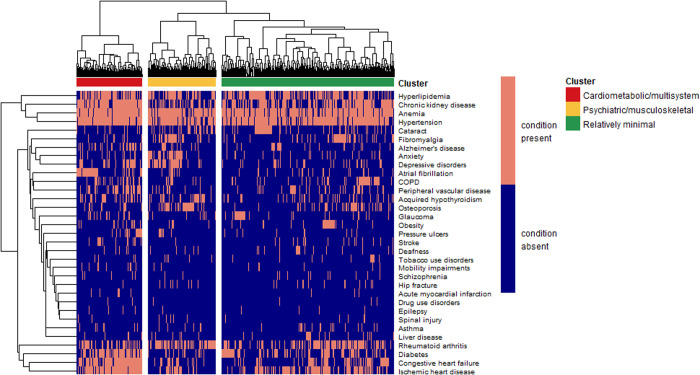
Heatmap diagram of multimorbidity patterns identified in older adults diagnosed with multiple myeloma, SEER-Medicare 2007–2017. Columns represent patients and rows represent chronic conditions. Green block: minimal multimorbidity; Yellow block: psychiatric and musculoskeletal multimorbidity; Red block: cardiometabolic and multisystem multimorbidity. In the heatmap, the presence of a chronic condition is indicated with light red, while the absence of that condition is indicated by dark blue. COPD; chronic obstructive pulmonary disease.

Three clusters of patients were separated based on the patterns of their baseline chronic health conditions (Table 3). We determined the three-cluster solution as optimal guided by explorations of different statistical clustering solutions and clinician feedback (see S1 Text). S2 Table shows proportions, O/E ratios and exclusivity for all the chronic conditions in each cluster, while Table 3 summarizes this information for the conditions with the highest of these values. The first cluster (54.3% of entire study cohort) comprised patients with high O/E ratios and exclusivity for only obesity, glaucoma, tobacco use disorder and liver disease. In this cluster, the prevalence of all other conditions was comparable to that observed in the general study cohort. We labeled this cluster relatively minimal multimorbidity to accurately reflect its distribution and enhance interpretability. The second cluster (21.5%) consisted of persons with patterns of psychiatric, pain-related and musculoskeletal conditions including anxiety, osteoporosis, depressive disorders, fibromyalgia, and fractures, as well as drug use disorder. The third cluster (20.7%) included individuals with a high multimorbidity burden featuring primarily cardiovascular conditions, and a wide array of respiratory, endocrine, and organ-specific conditions (Fig 2). The prevalence of anemia was high across all the patterns (84%−94%). Results obtained using the flexible beta algorithm were similar (S3 Table).

**Table 3:** Results from hierarchical cluster analysis identifying patterns of multimorbidity and defining conditions among older adults at time of diagnosis with multiple myeloma.

Multimorbidity pattern	Defining chronic conditions	Count	Prevalence (%)	O/E Ratio	Exclusivity (%)	O/E Ratio 95% CI
**Relatively minimal multimorbidity** **(n = 6,481)**	Obesity	806	12.4	1.15	65.0	1.08–1.24
	Glaucoma	924	14.3	1.14	64.4	1.07–1.22
	Tobacco use disorders	463	7.1	1.12	63.1	1.02–1.23
	Liver disease	535	8.3	1.10	62.1	1.01–1.20
**Psychiatric and musculoskeletal multimorbidity** **(n = 2,563)**	Anxiety	894	34.9	2.55	56.7	2.38–2.72
	Osteoporosis	924	36.1	2.01	44.7	1.88–2.14
	Depressive disorders	1099	42.9	1.94	43.2	1.83–2.06
	Drug use disorders	106	4.1	1.72	38.4	1.41–2.09
	Spinal injury	88	3.4	1.48	33.0	1.19–1.82
	Schizophrenia and other psychotic disorders	138	5.4	1.36	30.3	1.14–1.61
	Hip/Pelvic fracture	138	5.4	1.36	30.2	1.14-1.60
	Fibromyalgia	772	30.1	1.34	29.9	1.25–1.44
	Rheumatoid arthritis	1671	65.2	1.32	29.3	1.25–1.38
	Cataract	710	27.7	1.28	28.5	1.19–1.38
**Cardiometabolic and multisystem multimorbidity** **(n = 2,467)**	Acute myocardial infarction	201	8.1	2.93	62.8	2.54–3.37
	Atrial fibrillation	1152	46.7	2.56	54.8	2.41–2.71
	Pressure ulcers	567	23.0	2.23	47.7	2.05–2.42
	Stroke	406	16.5	2.15	46.1	1.95–2.37
	Congestive heart failure	1951	79.1	2.09	44.7	1.99–2.18
	Mobility impairments	228	9.2	1.93	41.5	1.69–2.20
	Peripheral vascular disease	861	34.9	1.84	39.5	1.72–1.97
	Alzheimer’s disease and related dementias	686	27.8	1.74	37.2	1.61–1.87
	Schizophrenia and other psychotic disorders	168	6.8	1.72	36.9	1.47–2.00
	Ischemic heart disease	2036	82.5	1.66	35.5	1.58–1.73
	Diabetes	1438	58.3	1.60	34.3	1.52–1.69
	Epilepsy	81	3.3	1.50	32.1	1.19–1.86
	Chronic kidney disease	1938	78.6	1.40	30.0	1.34–1.47
	Depressive disorders	709	28.7	1.30	27.9	1.21–1.40
	Acquired hypothyroidism	631	25.6	1.28	27.4	1.18–1.38
	Chronic obstructive pulmonary disease	604	24.5	1.23	26.5	1.14–1.34
	Hip/Pelvic fracture	120	4.9	1.23	26.3	1.02–1.47
	Hyperlipidemia	1727	70.0	1.17	25.0	1.11–1.22

**Notes.** O/E Ratio: observed/expected ratio, CI: confidence interval. Defining conditions presented in this table were selected based on an O/E Ratio > 1, exclusivity > 25% and 95% CI that excludes the null. O/E ratios were calculated by dividing the prevalence of the condition in the cluster by the prevalence in the overall cohort. Exclusivity was calculated by dividing the number of patients with the condition in the cluster by the total number of individuals with the condition.

### Demographic characteristics of multimorbidity patterns

Patients in the cardiometabolic and multisystem multimorbidity pattern were the oldest group at the time of multiple myeloma diagnosis (median age, 79 years), and were more likely to live in the highest poverty census tract (21%) compared to other groups (Table 1). Relative to the two other patterns, this pattern also included a greater proportion of Hispanic individuals (6.9%) and men (58.1%). The psychiatric and musculoskeletal multimorbidity pattern comprised the largest proportion of women (56%) and non-Hispanic White (80.2%), and the lowest proportion of non-Hispanic Black individuals (10.6%) compared to the other two patterns. Those with relatively minimal multimorbidity were the most likely to be married.

## Discussion

This study identified three multimorbidity patterns in an older adult population of Medicare beneficiaries with newly diagnosed multiple myeloma: 1) relatively minimal multimorbidity featuring minor burden of most comorbid conditions; 2) psychiatric and musculoskeletal multimorbidity; and 3) cardiometabolic and multisystem multimorbidity. Women were overrepresented in the psychiatric and musculoskeletal multimorbidity pattern, and people with low socioeconomic resources were overrepresented in the cardiometabolic and multisystem multimorbidity pattern. By classifying patterns of multimorbidity, we can assess the co-occurrence and burden of specific chronic health conditions that may impact a patient’s health beyond their cancer.

It is important to determine cancer patients’ complete health status to ensure that cancer management and treatment practices will not conflict with those of other chronic conditions. Our data-driven approach provides a clear visualization of the types of chronic conditions that consistently group together in this patient cohort. In this patient population, nearly all individuals had at least two conditions at time of diagnosis. This saturation of the currently accepted definition of multimorbidity is expected, given the older adult study population. Beyond counting the number of chronic conditions, different approaches are needed to better define and understand the nature and impact of multiple chronic conditions.

Making treatment decisions that account for the complexity of having multiple chronic conditions is often challenging. The clinical guidelines for multiple myeloma recommend that having no comorbidities and being <70 years old qualifies patients to receive the standard of care drug therapies followed by stem cell transplant [39]. This potentially reduces access to standard therapies for large portions of patients since multimorbidity is a prevailing health problem in older adults. As individuals with multimorbidity are not often included in clinical trials [40], guidelines for patients with specific combinations of chronic conditions are not available. Considering that various treatments for multiple myeloma cause toxic side effects [41], the challenge of deciding on optimum treatment modalities for patients with multimorbidity remains. Developing treatment protocols and clinical trials that account for patient multimorbidity starts with identifying common multimorbidity patterns in generalizable cohorts of older adults. Such knowledge can aid in further exploration of how multimorbidity may be associated with treatment choice, targeting treatment options for patients with certain chronic conditions and lastly, improving long-term health outcomes for older individuals with multiple myeloma.

Despite analytic variations, the multimorbidity patterns observed in the present study are similar to those reported in other studies of older adults [42] and specifically with multiple myeloma [21]. The prevalent patterns of multimorbidity identified in the study of US veterans using latent class methods, included cardiovascular and metabolic disease, psychiatric and substance use disorders, chronic lung disease, and multisystem impairment [21]. In a systematic literature review examining patterns of multimorbidity in cancer survivors, cardiometabolic patterns and the clustering of mental health conditions with musculoskeletal disorders have been observed as well [43].

The highest percentage of patients in our study belonged to the minimal multimorbidity pattern, marked by the low prevalence of most chronic conditions. In studies that use similar data-driven methods to partition chronic conditions in older adults, clusters reflective of individuals with low multimorbidity have emerged [21,44]. The predominance of obesity and tobacco use disorder in this pattern may point to their roles as risk factors for various chronic diseases. Interestingly, ocular comorbidities such as glaucoma observed in the pattern have been previously documented in patients with multiple myeloma, and may relate to the deposition of monoclonal proteins in the eye lens [45].

The second most prevalent pattern was highlighted by the presence of concurrent psychiatric, musculoskeletal, and pain-related conditions. The co-occurrence of mental and pain-related disorders in older adults has been extensively documented in the literature [46]. This includes prior findings where mental disorders are their own standalone multimorbidity pattern in patients with multiple myeloma treated at the VA [21], and cases where these conditions cluster with musculoskeletal and pain-related disorders in other populations [47]. These combinations of conditions potentially share complex biological pathways as they have similar neural mechanisms [48]. The majority of individuals within this group were women, and scientific literature suggests that women experience and report more of these conditions compared to men [49]. This multimorbidity pattern represents a patient group that may have difficulty in completing treatment and management plans due to experiencing functional limitations and mental health issues, and that may be at risk for worse health outcomes [50].

The third most common multimorbidity pattern was characterized by high multimorbidity featuring cardiovascular, endocrine, respiratory, and other systemic impairments. This pattern included the highest proportion of people living in an area with poverty levels ≥20%, which is consistent with research that shows associations between poverty and multimorbidity [29]. The study of older US veterans with multiple myeloma, which identified patterns using latent class analysis, identified a similar pattern featuring cardiovascular and metabolic conditions, as well as a separate multisystem impairment pattern defined by conditions impacting various body systems [21]. In other investigations of multimorbidity patterns in older adults, conditions affecting the cardiovascular and endocrine systems tend to cluster together as well [42]. The conditions clustered within this group are also reflective of those that are highly prevalent in nursing home residents [51]. This pattern reflects a patient subgroup that experiences an overwhelmingly high burden of serious chronic health conditions that may likely impact cancer prognosis and the overall health care decision-making process. Future research should explore this subgroup to determine their healthcare utilization, treatment modalities, and health outcomes.

### Strengths and limitations

This study is the first to our knowledge that explored patterns of multimorbidity in a population-based sample of older adult Medicare beneficiaries diagnosed with multiple myeloma and examined how these patterns differ according to select patient characteristics. The study offers a large sample size and is sufficiently powered to observe accurate estimates in the patient cohort. Our findings are generalizable to adults aged ≥65 years with newly diagnosed multiple myeloma in the US with fee-for-service Medicare, an age group that makes up two-thirds [5] of all newly diagnosed cases. The findings should be interpreted in the context of a few limitations. It is unknown whether examining individuals with other forms of Medicare, including Medicare Advantage, may produce similar results. While the CCW algorithms have been validated, some have the potential for misclassifying conditions that do not require frequent health care utilization [52]. Although the claims-based diagnosis codes cannot be verified using other data sources, this misclassification is likely non-differential with regard to myeloma status. Further, the identified multimorbidity patterns are in agreement with existing literature and studies using different approaches, so the potential for differential misclassification is low. The HCA method is exploratory and may produce different results depending on which clustering algorithms are used. However, we observed comparable results upon applying a different clustering algorithm. In addition to variations in adopted methods, the chronic disease compositions of the multimorbidity patterns may also differ due to the underlying study population, data sources and structures, and the specific chronic conditions that serve as the input for the partitioning method [53]. Nevertheless, most findings from this study (specifically the multimorbidity patterns we observed) are in agreement with previous research [21,42].

## Conclusions

In summary, patterns of multimorbidity identified among a population-based sample of Medicare beneficiaries diagnosed with multiple myeloma were similar to those identified in other older adult population settings, including those with multiple myeloma and other cancers. Cardio-metabolic, musculoskeletal and psychiatric conditions are three major patterns of multimorbidity frequently observed in these populations [42]. Our study offers further insights on sociodemographic factors associated with these patterns, in addition to confirming that these patterns are replicated in a generalizable population of older adults with multiple myeloma. In particular, our study has further highlighted the co-occurrence of psychiatric conditions with musculoskeletal disorders in this patient population, as well as observed a group with minimal multimorbidity. Our results may help clinicians be cognizant of certain patient groups that may be more likely to present with certain patterns of conditions to promote greater attention to their unique care needs and how this may influence treatment choice, response, and prognosis. The findings from this study also provide insights for future studies that could explore tailoring treatment recommendations informed by baseline conditions in older adults newly diagnosed with multiple myeloma to improve patient outcomes.

## Supporting information

S1 Table Modifications made to the chronic conditions in the Centers for Medicare and Medicaid Services Chronic Conditions Data Warehouse. (DOCX)

S2 Table Prevalence, observed/expected ratios and exclusivity of all chronic conditions per multimorbidity pattern derived from the hierarchical cluster analysis using the Ward algorithm. (DOCX)

S3 Table Prevalence, observed/expected ratios and exclusivity of all chronic conditions per multimorbidity pattern derived from the hierarchical cluster analysis using the flexible beta algorithm set to –0.5. (DOCX)

S1 Text Supplemental technical appendix. (DOCX)
