# Supplementary material for: Patterns of multimorbidity in older adults with multiple myeloma: An analysis of SEER-Medicare
Source: PLoS One. 2025 Aug 20;20(8):e0330331. doi: 10.1371/journal.pone.0330331 (PMC12367123; doi:10.1371/journal.pone.0330331)
Supplement: S1 Table — (DOCX) [file pone.0330331.s001.docx]

**S1 Table.** Modifications made to the chronic conditions in the Centers for Medicare and Medicaid Services Chronic Conditions Data Warehouse

| **Original conditions** | **Source** | **Combined names** |
| --- | --- | --- |
| Depression | 27 CCW Chronic Conditions (1999–2021) | Depressive Disorders |
| Depressive Disorders | 40 Other Chronic Health, Mental Health, and Potentially Disabling Conditions |  |
| Alzheimer's Disease | 27 CCW Chronic Conditions (1999–2021) | Alzheimer's Disease and Related Disorders or Senile Dementia |
| Alzheimer's Disease and Related Disorders or Senile Dementia | 27 CCW Chronic Conditions (1999–2021) |  |
| Schizophrenia | 40 Other Chronic Health, Mental Health, and Potentially Disabling Conditions | Schizophrenia and Other Psychotic Disorders |
| Schizophrenia and Other Psychotic Disorders | 40 Other Chronic Health, Mental Health, and Potentially Disabling Conditions |  |
