# Supplementary material for: Patterns of multimorbidity in older adults with multiple myeloma: An analysis of SEER-Medicare
Source: PLoS One. 2025 Aug 20;20(8):e0330331. doi: 10.1371/journal.pone.0330331 (PMC12367123; doi:10.1371/journal.pone.0330331)
Supplement: S3 Table — (DOCX) [file pone.0330331.s003.docx]

**S3 Table.** Prevalence, observed/expected ratios and exclusivity of all chronic conditions per multimorbidity pattern derived from the hierarchical cluster analysis using the flexible beta algorithm set to –0.5.

|  | Relatively minimal | | | Psychiatric and musculoskeletal | | | Cardiometabolic and multisystem | | |
| --- | --- | --- | --- | --- | --- | --- | --- | --- | --- |
|  | **Prevalence (%)** | **O/E Ratio** | **Exclusivity (%)** | **Prevalence**  **(%)** | **O/E Ratio** | **Exclusivity (%)** | **Prevalence (%)** | **O/E Ratio** | **Exclusivity (%)** |
| Anemia | 86.1 | 0.99 | 32.8 | 84.6 | 0.97 | 45.7 | 93.9 | 1.08 | 21.4 |
| Chronic kidney disease | 42.3 | 0.76 | 25.0 | 53.6 | 0.96 | 44.9 | 84.7 | 1.51 | 30.0 |
| Liver disease | 2.5 | 0.34 | 11.3 | 10.5 | 1.40 | 66.0 | 8.6 | 1.14 | 22.7 |
| Acute myocardial infarction | 0.3 | 0.12 | 4.1 | 2.4 | 0.85 | 40.0 | 7.8 | 2.82 | 55.9 |
| Atrial fibrillation | 9.8 | 0.54 | 17.8 | 16.3 | 0.89 | 42.0 | 37.0 | 2.03 | 40.2 |
| Congestive heart failure | 13.1 | 0.34 | 11.4 | 35.9 | 0.95 | 44.5 | 84.0 | 2.22 | 44.0 |
| Hypertension | 75.3 | 0.90 | 29.8 | 84.3 | 1.01 | 47.3 | 96.8 | 1.15 | 22.9 |
| Ischemic heart disease | 24.0 | 0.48 | 16.0 | 53.0 | 1.06 | 49.9 | 85.6 | 1.72 | 34.1 |
| Peripheral vascular disease | 5.8 | 0.31 | 10.2 | 20.0 | 1.06 | 49.7 | 38.3 | 2.02 | 40.1 |
| Stroke | 2.0 | 0.27 | 8.9 | 8.6 | 1.13 | 53.1 | 14.7 | 1.92 | 38.1 |
| Diabetes | 29.4 | 0.81 | 26.7 | 32.7 | 0.90 | 42.2 | 57.0 | 1.57 | 31.1 |
| Hyperlipidemia | 50.3 | 0.84 | 27.8 | 61.7 | 1.03 | 48.3 | 72.0 | 1.20 | 23.9 |
| Acquired hypothyroidism | 19.8 | 0.99 | 32.8 | 18.6 | 0.93 | 43.7 | 23.6 | 1.18 | 23.5 |
| Obesity | 3.9 | 0.36 | 11.9 | 13.0 | 1.20 | 56.5 | 17.1 | 1.59 | 31.6 |
| Alzheimer’s disease and related dementias | 3.8 | 0.24 | 7.8 | 21.0 | 1.31 | 61.6 | 24.7 | 1.54 | 30.6 |
| Anxiety | 3.7 | 0.27 | 8.9 | 21.1 | 1.54 | 72.3 | 12.9 | 0.95 | 18.8 |
| Depressive disorders | 7.4 | 0.33 | 11.0 | 29.5 | 1.33 | 62.7 | 29.2 | 1.32 | 26.3 |
| Epilepsy | 1.0 | 0.48 | 15.9 | 2.6 | 1.17 | 54.8 | 3.2 | 1.48 | 29.4 |
| Schizophrenia and other psychotic disorders | 1.3 | 0.33 | 11.0 | 4.8 | 1.22 | 57.4 | 6.3 | 1.59 | 31.6 |
| Asthma | 4.0 | 0.52 | 17.4 | 8.1 | 1.05 | 49.5 | 12.8 | 1.67 | 33.1 |
| Chronic obstructive pulmonary disease | 11.6 | 0.59 | 19.5 | 14.2 | 0.71 | 33.6 | 46.9 | 2.37 | 47.0 |
| Fibromyalgia | 11.3 | 0.51 | 16.8 | 34.2 | 1.52 | 71.6 | 13.1 | 0.59 | 11.6 |
| Hip/Pelvic fracture | 1.3 | 0.32 | 10.7 | 5.9 | 1.49 | 70.2 | 3.8 | 0.96 | 19.0 |
| Mobility impairments | 0.9 | 0.19 | 6.2 | 5.8 | 1.22 | 57.5 | 8.7 | 1.83 | 36.4 |
| Osteoporosis | 15.7 | 0.88 | 29.0 | 21.5 | 1.20 | 56.3 | 13.3 | 0.74 | 14.7 |
| Rheumatoid arthritis | 36.2 | 0.73 | 24.2 | 59.7 | 1.21 | 56.6 | 47.7 | 0.96 | 19.1 |
| Spinal injury | 1.0 | 0.43 | 14.2 | 3.3 | 1.43 | 67.4 | 2.1 | 0.92 | 18.4 |
| Cataract | 26.0 | 1.20 | 39.8 | 21.0 | 0.97 | 45.6 | 16.0 | 0.74 | 14.6 |
| Glaucoma | 16.1 | 1.29 | 42.9 | 9.8 | 0.79 | 36.9 | 12.7 | 1.02 | 20.2 |
| Deafness | 2.1 | 0.30 | 9.8 | 10.9 | 1.53 | 72.0 | 6.5 | 0.92 | 18.2 |
| Drug use disorders | 0.8 | 0.34 | 11.2 | 3.5 | 1.45 | 68.1 | 2.5 | 1.04 | 20.7 |
| Tobacco use disorders | 6.0 | 0.94 | 31.2 | 6.1 | 0.95 | 44.7 | 7.7 | 1.21 | 24.1 |
| Pressure ulcers | 2.7 | 0.26 | 8.6 | 10.5 | 1.02 | 47.9 | 22.6 | 2.19 | 43.5 |

**Notes**. Conditions with prevalence >2% were included. O/E ratios were calculated by dividing the prevalence of the condition in each cluster by its prevalence in the overall cohort. Exclusivity was calculated by dividing the number of patients with the condition in the cluster by the total number of individuals with the condition. Chronic conditions with an O/E >1 and exclusivity >25% were used to define patterns. Conditions are ordered according to their conceptual definition (e.g., cardiovascular).
