# Supplementary material for: Patterns of multimorbidity in older adults with multiple myeloma: An analysis of SEER-Medicare
Source: PLoS One. 2025 Aug 20;20(8):e0330331. doi: 10.1371/journal.pone.0330331 (PMC12367123; doi:10.1371/journal.pone.0330331)
Supplement: S1 Text — (DOCX) [file pone.0330331.s004.docx]

**S4 Text.** Supplemental technical appendix

**Notes on the chronic conditions from the Chronic Conditions Data Warehouse (CCW)**

We combined similar conditions into one variable when overlapping algorithms were used for their derivation or when all individuals with one condition were nested in another condition (e.g., “depressive disorders” and “depression” were combined into one condition) (See S1 Table). The CCW chronic condition flags comprise a set of four categories indicating whether a beneficiary met the criteria for having that condition throughout the algorithm lookback period. The flags require beneficiaries to satisfy claims criteria (minimum number and type of claims with the proper diagnosis codes occurring within the specified time period) and coverage criteria (fee-for-service [FFS] Part A and Part B coverage during the entire specified time period). The four available codes and their values are: 0-Beneficiary did not meet claims criteria nor have sufficient FFS coverage, 1-Beneficiary met claims criteria but did not have sufficient FFS coverage, 2-Beneficiary did not meet claims criteria but had sufficient FFS coverage, 3-Beneficiary met claims criteria and had sufficient FFS coverage. We included only beneficiaries with values of 2 or 3 for all conditions. Including those with values of 0 or 1 in some conditions introduces missingness in the chronic condition distribution, which would have disrupted the HCA procedure. We opted to exclude both values 0 and 1 because for the 0 category, there may have been some individuals who had the given condition (but not captured in the data). Including those with 1 (and not 0) would bias the cohort distribution towards those that have the condition.

**Description of methods used to select an optimal number of multimorbidity clusters**

We used the NbClust procedure in R, along with examination of exploratory graphics including dendrograms, elbow and silhouette plots to determine an optimal number of clusters to be extracted from the hierarchical cluster analysis. The NbClust process evaluates the clustering structure by comparing it with other clustering schemes resulting from the same algorithm and ranks the preferred cluster number according to a majority rule. We conducted this process for the training, validation, and complete datasets and though NbClust determined two to be the best number of clusters by most of the statistical parameters, the graphs pointed towards three-four potentially being optimal. We explored the 2, 3, and 4-cluster solution and found that a 4-cluster solution did not provide additional information regarding the content of the fourth cluster.

**NbClust agreement**

Among the 23 indices that were used to classify the correct number of clusters within the dataset:

8 proposed 2 as the best number of clusters

5 proposed 3 as the best number of clusters

6 proposed 5 as the best number of clusters

2 proposed 8 as the best number of clusters

2 proposed 9 as the best number of clusters

**NbClust graph outputs**

The Hubert index is a graphical method of determining the number of clusters. In the plot of Hubert index, we seek a significant knee that corresponds to a significant increase of the value of the measure i.e the significant peak in Hubert index second differences plot. The D index is a graphical method of determining the number of clusters. In the plot of D index, we seek a significant knee (the significant peak in Dindex second differences plot) that corresponds to a significant increase of the value of the measure.

**
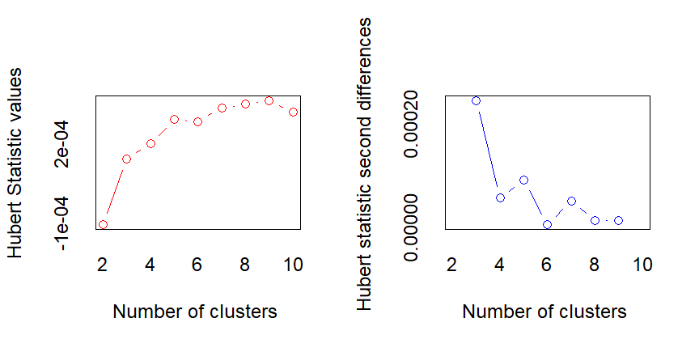
***
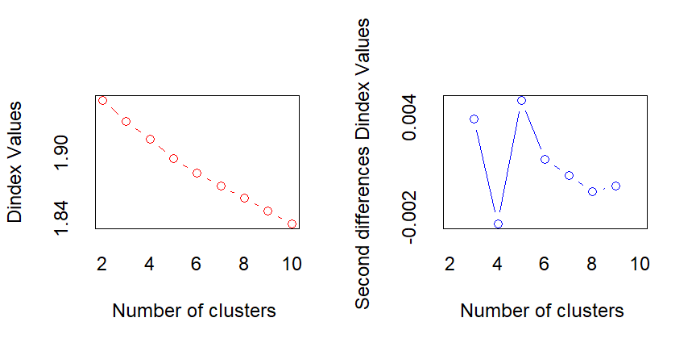
*
